# The revival of intrauterine insemination: evidence-based data have changed the picture

**Published:** 2017-09

**Authors:** Willem Ombelet

**Affiliations:** Editor-in-Chief; Genk Institute for Fertility Technology, ZOL Hospitals, Schiepse Bos 6, 3600 Genk, Belgium; Hasselt University, Department of Physiology, Martelarenlaan 42, 3500 Hasselt, Belgium

**Keywords:** artificial insemination, assisted reproduction, intrauterine insemination, IUI, NICE guidelines, semen quality, unexplained infertility

## Abstract

According to a number of high quality studies intrauterine insemination (IUI) with homologous semen should be the first choice treatment in case of unexplained and moderate male factor subfertility. IVF and ICSI are clearly over-used in this selected group of infertile couples.

The limited value of IUI in infertility treatment as mentioned in the 2013 NICE guidelines was surely a premature statement and should be adapted to the actual literature.

More evidence-based data are becoming available on different variables influencing the success rates after IUI. It can be expected that these findings may lead to a better understanding and use of IUI in the near future.

Assisted reproductive technologies (ART) are considered as an established therapy for the treatment of infertility in a multitude of clinical conditions. It embraces a wide scope of techniques of which intrauterine insemination (IUI), in-vitro fertilization (IVF) and intra-cytoplasmic sperm injection (ICSI) are most popular.

Because IUI is a simple and non-invasive technique it can be performed without expensive infrastructure with a reasonable success rate within three or four cycles. It is a safe and easy treatment with minimal risks and monitoring. Subsequently IUI has a good couple compliancy (low drop-out rate) and a very low risk for complications such as OHSS (ovarian hyperstimulation syndrome).

Nevertheless, the use of IUI as a first-line treatment in case of unexplained and mild/moderate male infertility remained very controversial until very recently. This was due to a lack of prospective randomized trials and large prospective cohort studies caused by the low budget linked to IUI when compared to the budget associated with other methods of assisted reproduction such as IVF and ICSI. Large multicentre trials organised by the pharmaceutical industry are not available in the IUI scene.

Based on the results of an ESHRE workshop in 2009 IUI was considered to be a poor substitute for IVF and associated with a significant rate of high-order multiple births. The recommendations were made in the absence of proper trials and absent live birth data (ESHRE, 2009). It was also not mentioned that the high rate of multiple pregnancies was mostly seen outside of Europe and caused by using high doses of gonadotrophins.

The NICE guidelines (NICE, 2013) recommended that in case of unexplained infertility failed expectant management for up to two years should be followed directly by IVF treatment suggesting that IUI has a very limited value in infertility care. It’s well known that the NICE guidelines were being constructed by using the data of a few studies with obvious shortcomings and not taking into account the HFEA data showing an UK avarage pregnancy rate of 13 % per cycle for IUI in 2011 and 2012 (Bhattacharya et al., 2008; Reindollar et al., 2010; Wordsworth et al., 2011; HFEA, 2015). Despite the guidelines surveys performed in the UK showed that 96% of fertility clinics continued to offer IUI - despite the NICE recommendations (Kim et al., 2015; Nandi et al., 2015; Bahadur et al., 2016).

Since then, a number of excellent randomized trials have been published supporting the value of IUI. In a multicentre randomised non-inferiority trial in the Netherlands, the effectiveness of IVF with single embryo transfer or IVF in a modified natural cycle was compared with the effectiveness of IUI-OS (ovarian stimulation) with a healthy live birth as the main outcome parameter (Bensdorp et al., 2015). IUI-OS seemed to be non-inferior compared to the two alternative strategies of IVF, with a reasonably low multiple birth rate. Investigating the direct health care costs in the same cohort of patients IUI turned out to be the most cost-effective strategy for heterosexual couples with mild male factor or unexplained infertility with a poor prognosis of becoming pregnant through normal coitus (Tjon-Kon-Fat et al., 2015).

On the occasion of the annual meeting of ESHRE (European Society of Human Reproduction and Embryology) in Geneva, July 2017, the results of two randomized controlled trials (RCT) on IUI and unexplained infertility were presented. Cindy Farquhar showed the results of an RCT in which 201 couples with 3-4 years unexplained infertility were randomised to receive three cycles of IUI or expectant management (Farquhar et al., 2017a, 2017b). A live birth rate of 31% with IUI and 9% with expectant management was observed, a three-fold difference in outcome. In another RCT performed in the Netherlands stimulated IUI with clomiphene citrate turned out to be first-line therapy compared to low dose FSH (Danhof et al., 2017).

According to the above mentioned studies it’s obvious that we are over-using IVF to treat unexplained infertility. Anno 2018 evidence-based data clearly indicate that promoting IVF and ICSI to result in pregnancy “as quick as possible” ignores the advantages of IUI completely in case of unexplained and mild male factor infertility.
